# What makes a good research invitation for patient and public participants in core outcome set studies: a UK-based consensus meeting to develop guidance

**DOI:** 10.1136/bmjopen-2025-111017

**Published:** 2026-08-18

**Authors:** Heather Barrington, Rebecca Craven, Paula R Williamson, Bridget Young

**Affiliations:** 1 Health Data Science Department, University of Liverpool, Liverpool, UK

**Keywords:** STATISTICS & RESEARCH METHODS, Patient Participation, Research Design

## Abstract

**Abstract:**

**Background:**

It is crucial that the outcomes used in clinical effectiveness studies are relevant to patients. This relevance can be assured by patients participating with other key stakeholders in core outcome set (COS) studies, yet recruiting patients to such studies can be challenging.

**Objectives:**

As no guidance currently exists to help COS developers create good COS invitations for patients, which are usually the first documents patients see when invited to participate in COS studies, the Good Research Invitation Study aimed to produce such guidance.

**Design:**

Consensus meeting to agree on guidance for COS developers designing research invitations.

**Setting and participants:**

Informed by findings from our previous qualitative study of patient and public opinions on COS study invitations, our team, which included a patient research partner, produced an initial set of candidate recommendations. An online consensus meeting was organised in the UK with 13 participants including patient research partners with and without COS study experience, COS developers, a representative from an ethics committee and experts in diversity, marketing and science communication. Through a process of discussion and voting, the initial set of recommendations was refined.

**Results:**

From the initial 35 recommendations, consensus meeting participants agreed a final set of 22 recommendations. Some initial recommendations were removed as they were beyond the scope, while others were merged. The finalised recommendations focused on producing and user testing invitations, reaching the audience, engaging potential COS participants and considering what they might find appealing or off-putting, explaining and creating interest in the study, keeping the invitation simple and understandable, considering the amount of information to include and how to present it, alongside ensuring accessibility. Discussion during the meeting highlighted the importance of improving the accessibility of all participant-facing resources in COS studies, not just the invitations.

**Conclusion:**

Through consensus, a set of recommendations has been agreed on for use by COS developers to enhance study invitations for patients. Further testing and refinement of the recommendations will improve their utility.

**STRENGTHS AND LIMITATIONS OF THIS STUDY**

- The consensus meeting included a diverse group of stakeholders including patient research partners, those with expertise in core outcome set (COS) development, communication, ethics, diversity and marketing; however, representation was limited in the under 40s age ranges.
- Consensus meeting study design and deliberation were informed by previous qualitative research with patients and the public who gave their views on real examples of COS invitations.
- The guidance was developed with predominantly UK-based participants; factors such as cultural differences and variation in accessibility issues may affect the relevance of the guidance beyond the UK.
- Evaluation of the guidance outside of the UK would help in understanding its transferability outside the UK.

## Introduction

 Randomised controlled trials are considered the gold standard for evaluating the effectiveness and safety of healthcare interventions. However, when trials lack relevance to patients and the public, or have methodological flaws and inefficiencies, their impact can be diminished or lost.^1–7^ One way to help ensure a trial’s findings are relevant to future patients is by selecting patient-important outcomes, although the lack of patient relevance in outcome selection is a widespread problem in trials. A systematic review of outcomes in neonatal trials^8^ showed that, except for survival, most neonatal trials only reported outcomes relating to diagnoses in the neonatal admission period, disregarding outcomes relating to the impacts of prematurity in childhood and beyond. In rheumatoid arthritis, fatigue was an outcome not routinely measured in trials, yet it was found to be important to patients.^9^ Noble *et al*^10^ surveyed people living with epilepsy and their carers to identify patient important outcomes, comparing these to those recommended by health professionals who had participated in International League Against Epilepsy (ILAE) round table discussions to identify the most important outcomes for epilepsy research. While there was agreement on the outcomes identified by the ILAE, further domains were identified by patients and carers: depression, anxiety and independence/need for support.

Clinical trial outcomes have traditionally been selected by researchers and yet evidence indicates patients may prioritise different outcomes to researchers.^9 11 12^ Patient and public involvement (PPI, where patients collaborate with researchers on the design, delivery and dissemination of research) in clinical trials is considered essential to ensure the research meets the needs, priorities and preferences of those they serve. PPI throughout the research process is recommended, including in outcome selection.^13–15^ However, available evidence suggests that their involvement in outcome selection is constrained.^8 16^ For example, one study indicated that patients had been given little choice in the outcomes to select from.^17^ Brett *et al*^18^ have called for resources to support patient research partners (patients who collaborate with researchers in the design, delivery and dissemination of research) in outcome selection and similar tools have been requested to help trialists (researchers who design, run and analyse clinical trials) maximise how they engage patients in outcome selection.^17^ This suggests that current involvement processes require improvement. A consensus meeting prioritising methodological questions about PPI in trials identified ‘exploring PPI practices in selecting trial outcomes of importance to patients’ as a key question,^19^ further emphasising concerns in this field. Where PPI in outcome selection entails just one or two patient research partners providing their views on patient important outcomes for a clinical trial, it will be unclear whether the outcomes selected are appropriate to a wider, more diverse, patient population. Wider representation of patients is needed to be confident that outcomes selected are patient important outcomes.

Alongside the lack of patient centricity in outcome selection are concerns about heterogeneity in clinical trial outcomes and selective reporting bias, both of which create substantial waste in clinical trials, delaying translation of research into practice. Numerous systematic reviews of outcomes in clinical trials have identified the lack of standardisation in outcome selection, such that when trials are completed, their results cannot be compared and combined in systematic reviews and meta-analyses as the trials have measured different outcomes.^20–23^ Similarly, there is evidence of reporting bias, with clinical trials reporting only some of the outcomes they collected.^24^

Core outcome sets (COS) have been developed to address these three challenges in clinical trials. COS are standardised sets of outcomes that should be measured and reported, as a minimum, in all clinical trials in a specific health area or intervention.^25^ The use of COS is endorsed by numerous organisations,^26^ most recently the World Health Organisation (WHO) in its Guidance for Best Practices in Clinical Trials.^27^ COS are often agreed through studies that include Delphi surveys and consensus meetings. Patients and the public are considered key stakeholders in COS development, alongside others such as clinicians or health researchers.^28^ In the context of COS development, PPI and patient participation are sometimes conflated and it is important to distinguish the two. When patients input to COS studies by contributing data to inform outcome identification and prioritisation, we refer to this as patient participation. This is distinct from the PPI we referred to earlier, where patients can input to COS studies by collaborating with researchers on the design, delivery and dissemination of these studies. By recruiting patient to participate in expansive COS surveys, COS avoid the limitations of traditional PPI for selecting patient important outcomes, particularly where the involvement accesses the views of very small numbers of patient research partners. Patients, however, may experience challenges with participating in COS surveys^29^ and concerns have been reported by COS developers about how best to include patients as participants in COS studies.^30^ While COS studies rarely report the denominator for individuals invited, a systematic review of patient participation in COS studies indicated that proportionately fewer patients participate in these studies compared with professional participants.^31^

Most COS studies use remote methods such as invitational emails and flyers to recruit participants into online Delphi surveys^31^ and the research invitation will usually be the first document that a potential patient participant in a COS study will see, before potentially progressing to read subsequent information such as the participant information sheet. It is, therefore, crucial that the invitation is understandable, accessible and engaging. To understand more about patient recruitment into COS studies, Barrington *et al*^32^ conducted the Good Research Invitation qualitative study with patients and the public to explore their perspectives on COS study invitations. Findings pointed to confusion among patients and the public about research terminology and to misunderstandings about the purpose of a COS. Patients and the public described feeling overwhelmed by the amount of information in invitations and wanted greater clarity about the benefits of COS participation. Fears of being scammed and nervousness about clicking on links and attachments in invitations were prominent, as were concerns about the inaccessibility of invitations.

As no guidance is available for COS developers to help them design good COS study research invitations for patients and the public, we aimed to produce such guidance via the Good Research Invitation Study (GRIS). GRIS comprised both the aforementioned qualitative study and a consensus meeting to inform the development of the guidance. Here, we report on the GRIS consensus meeting in which we brought together relevant expertise to help steer the development of the guidance and add credibility to it.^33 34^

## Methods

### Design

To develop and agree guidance through expert opinion, we conducted an online consensus meeting to agree guidance for COS developers on how best to design the initial study invitations sent to patients and the public for their studies (online supplemental appendix 1—Study Protocol). This meeting was held to enable a set of draft recommendations, derived from the findings of the earlier GRIS qualitative study,^32^ to be discussed, agreed, modified or rejected. The meeting benefited from the expertise of public research partners (with extensive PPI experience) and a range of other relevant professionals. Our study team included the expertise of the Lead and the PPI co-ordinator from the international Core Outcome Measures in Effectiveness Trials (COMET) Initiative, as well as the Co-Chair of COMET’s People and Public Participation Involvement and Engagement (PoPPIE) Working Group and a patient research partner who had taken part previously in a COS study. The protocol for this study was not registered, but the version approved by the Research Ethics Committee can be made available on request.

### Sampling and recruitment of participants

When planning the consensus meeting, the study team (HB, BY, PW and RC) aimed for sufficient numbers of participants to ensure diversity in expertise while also aiming for a manageable online discussion. We were clear on the need to include patient research partners with and without COS experience. Patient research partners with COS experience could reflect on how they were recruited or how COS teams they have collaborated with had addressed inviting patients. Those without COS experience could provide their perspectives on what it might be like to be invited into a COS study, an experience which will be unfamiliar to most patients. We included COS developers who were known to have a specific interest in patient participation and involvement, as they could reflect on their own COS patient recruitment experience and how practicable the guidance needed to be for future COS developers.

From our previous GRIS qualitative study, we knew that patients and the public can struggle to understand COS study invitations, so we were keen to include experts in the field of communication. Diversity issues clearly need to be addressed in COS study recruitment to ensure COS studies are accessible for all, so we chose to include a communication and marketing practitioner from an initiative focused on ethnicity in health research. We also wanted to ensure the guidance adequately reflected the importance of making invitations engaging yet not coercive. We felt an academic in marketing would bring expertise in the importance of creating engaging messages and audience segmentation, while members of ethics committees would bring knowledge of ethical principles and informed consent, which we recognised as crucial in ensuring the guidance protects the potential COS participant. We purposively sampled participants based on these categories. To ensure the guidance was patient focused, we included five patient research partners, but we also wanted a range of COS development experience so included three COS developers.

The patient research partners with COS experience were recruited through conversations with the two patient research partners on the international COMET Initiative PoPPIE Working Group. The patient research partners without COS experience, but who had wider experience in health research, were recruited by advert through PPI Groups in the Northwest of England. UK COS developers, who were also clinicians, were recruited through the COMET Initiative’s database of COS studies and were known to COMET as having a particular interest in patient participation in COS studies. They came from the Midlands, the southwest and the northwest of England. Academics with expertise in health research marketing and communication were recruited through universities in the southeast and northwest of England, identified through conversations with a local expert in marketing and links with the Trials Methodology Research Partnership in the UK. Ethics committee members were recruited through a university research ethics committee and through dissemination of the opportunity by the Health Research Authority. Communication and marketing practitioners from two health research organisations (one focused specifically on ethnicity in health research) were recruited via their respective organisations, from the Midlands and the southwest of England.

All potential participants were emailed inviting them to attend one online consensus meeting. Some were emailed directly (if they were already known to members of the research team), while others were identified through other researchers or through research organisations. If the potential participant was available and willing to participate, they were asked to complete a demographic characteristics form. Where participants were unavailable, we sought their advice on who else might be a suitable alternative and if our study team felt they would be appropriate, we invited them.

### Consensus meeting organisation and conduct

Drawing closely on the findings from the previous GRIS qualitative study, HB drafted the initial version of the guidance, with input from PW, BY and RC. Quotations from the focus groups were identified for the patient research partner RC to read at particular points during the consensus meeting to support some of the recommendations. Consensus meeting participants were sent the agenda, Zoom meeting instructions and list of recommendations (online supplemental appendix 2—list of recommendations) 3 weeks before the meeting and asked to familiarise themselves with the recommendations. A guide (online supplemental appendix 3) was produced for the facilitator of the consensus meeting (KW) and a preparatory meeting was held with her to finalise the details. Three hours were allocated for the consensus meeting, including breaks, and the Zoom communications platform was used to run and audio-record the meeting. Public research partners received a £75 high street voucher for participating in the study.

As noted above, the online consensus meeting was facilitated by KW, who was experienced in conducting consensus meetings while independent of the study. Voting was managed by a member of the COMET Initiative Team (SG), two note-takers took written notes in addition to the audio recording (NH & BY). The consensus meeting was conducted in English. KW led introductions and explained both the purpose of the meeting and how it would be conducted. Prior to attending the meeting, participants were advised in the participant information sheet that their participation implied consent. KW reminded the participants of this at the beginning of the consensus meeting and ground rules were agreed to ensure all participants felt able to share their views.

HB presented a summary of the findings of the GRIS qualitative study. KW then read each recommendation and RC read the identified quotations at relevant points. After each recommendation, participants were invited to comment on whether the recommendation was clear enough and whether they agreed to its inclusion. Through discussion, participants could either propose accepting the recommendation, rewording it, amalgamating it with other recommendation(s) or removing it. After discussion, if there were no objections to the action proposed by the group, the action was accepted. If there were disparities in opinions regarding the inclusion of the recommendation, voting took place (yes/no voting options) using anonymised voting in the Zoom platform. In line with recommended practice in consensus methodology, a predefined consensus cut-off point of 70% agreement in the group as a whole was used to determine whether consensus had been agreed to include the recommendation.^35^

At the end of the meeting, participants were informed that the recommendations would be revised in the light of the discussions, and the revised set of recommendations would be emailed to them for their review and comments. KW also invited meeting participants to send any additional post-meeting thoughts to HB for consideration. Participants were advised that an article would be published on the findings. The audio recording of the consensus meeting was transcribed by a professional transcription agency and then anonymised.

Following the meeting, we developed a spreadsheet containing the original wording of each recommendation and key points from the discussion of that recommendation. These key points included proposed word changes, suggested merging of recommendations, or agreed actions for the inclusion or removal of a recommendation. This spreadsheet was used to refine the guidance. The refined guidance was sent to participants for any further comments or suggestions. After this, typographical changes and minor adjustments for clarification were made. This article has been reported in accordance with the ACurate COnsensus Reporting Document (ACCORD).^36^

### Patient and public involvement statement

PPI in the design, delivery and dissemination of the consensus meeting was provided by a member of the public, a former COS study participant (RC) who had previously been involved as a patient research partner in the GRIS qualitative study. She collaborated on the design of the study protocol and participant-facing materials, the identification of recommendations and relevant quotes for the consensus meeting, presented the quotes during the consensus meeting and co-authored this article.

## Results

The online consensus meeting was held in November 2024. 22 individuals were invited to attend, 14 agreed to participate and 13 attended. Five were public research partners (two with COS experience), three were COS developers, one was a researcher with an ethics background and four were experts in communication, diversity or marketing. Regarding their ethnicity, 10 participants were White, two were Asian/Asian British, and one was Black/Black British/Caribbean or African. The remaining characteristics of these 13 participants are presented in table 1.

**Table 1:** Consensus meeting participant characteristics

Participant ID	Sex	Age group
1	Male	55–64
2	Female	45–54
3	Female	65–74
4	Female	35–44
5	Male	65–74
6	Female	45–54
7	Female	55–64
8	Female	65–74
9	Female	45–54
10	Female	45–54
11	Female	65–74
12	Male	65–74
13	Female	25–34

The meeting lasted for 2 hours and 44 min, including brief breaks. All 35 recommendations were discussed. Some participants, who were used to participating in COS studies, sought clarification on whether they were to prioritise the importance of the recommendations or whether they were to vote each recommendation in or out, they were advised to vote based on the latter. Online supplemental appendix 4 provides the original recommendation, key points from the discussion, agreed actions and data on voting.

From an initial set of 35 recommendations in the first version of the guidance, the final guidance contains 22 recommendations. Some of the original recommendations were identified as being beyond the scope of invitation guidance (for example, referring to study design or participant information sheets) while others were merged. Meeting participants advised re-ordering the recommendations to emphasise the sequence in which invitations would be designed and disseminated. The overall length and relatively large number of original recommendations was a concern to participants, so they suggested amendments to address this. The need to avoid unduly adding to the length of a potential COS invitation was also a key discussion point. Participants wanted the guidance to indicate an acceptable word length for invitations. Additional concerns raised were that improving the invitations would be of limited benefit if the remainder of the patient-facing resources in the COS study (for example the participant information sheet and the survey itself) were presented in such a way that made them challenging for patients and the public to access. The importance of encouraging COS developers to consider their patient population when using the guidance to design their invitations was highlighted, for example, rare disease communities may be more familiar with global participation and may therefore be more receptive to words such as ‘international’. The final agreed guidance is provided in box 1.

Box 1 **Final agreed guidance**

**Recommendations for creating initial invitations for core outcome set studies**

These recommendations aim to help core outcome set teams enhance the initial invitations or communications sent to potential participants in core outcome set studies (as distinct from the information sheets/materials for the subsequent informed consent process). The recommendations have been developed through a two-stage process: (1) focus groups with a diverse range of patients and the public from the UK who explored examples of previous COS invitations and (2) a consensus meeting attended by mostly UK-based participants with experience in core outcome set development, PPI, science communication, diversity and ethics committees. Variations in language, culture and systems across different countries may mean the recommendations need adapting for use outside the UK.

We use the term ‘public’ in this guidance to refer to patients, carers and members of the public who may be invited to participate in a core outcome set study.

**Producing and user testing your invitation**

1. Co-produce your study with patients and the public as a first step to ensure your invitation is engaging and understandable. Consider also consulting with communication experts or using writing enhancement tools.
2. User-test or pilot your study invitation with your target audience (ensuring they are a diverse group and not solely research-aware patient research partners). Do this even if patient research partners have been involved in the development of your invitation, as the public are often less familiar with medical or research terms than patient research partners.

**Reaching your audience**

1. Consider how you are going to disseminate the invitations to potential participants and the implications that this may have on a participant’s trust (ensuring you have the appropriate ethical approval for your recruitment approach). The public are suspicious of e-invitations and told us they have greater confidence if invitations are provided in trusted spaces, for example, a poster on the doctor’s clinic wall or in a community group setting, face-to-face invitations, an email/newsletter article from a patient organisation. Receiving a signed letter through the post might also address some of the concerns raised about cyber security.
2. Offer potential participants alternative methods to verify the authenticity of the invitation aside from web-links. The public are concerned about online safety. They told us that receiving an unsolicited email invitation from a stranger can be concerning, especially if they are asked to ‘click on a link’ for further information or register their interest. Even including logos from trusted organisations often fail to reassure as the public are aware that these can be easily copied. Likewise, some participants may have concerns about being scammed on phone calls.
3. Where invitations are being addressed directly to participants, make it clear how you came to have their name.

**Engaging potential participants**

1. Think about how meaningful study titles are to potential participants. If you choose to include a study title on the invitation, it should be simple and immediately resonate with them. Avoid using the study title if it contains an acronym—it requires people to decode it before they consider whether the study is relevant. The use of upper-case and lower-case letters or altered spelling of familiar words in study titles or acronyms further confuses the public.
2. Create interest in the study and maintain this so people read to the end of the invitation.
  - Use questions such as “Do you have xxxx (condition), or are you a carer for someone with this condition? Would you like to help improve the way xxxx treatments are developed?”
  - Get to the point of the invitation quickly and use language that demonstrates the research team are enthusiastic about the core outcome set study.
  - Ensure there is a clear reason for people to take part and emphasise the value of the patient/public perspective.
3. Think about what your audience will find appealing or off-putting
  - Avoid saying “help us improve future research.” While altruistic messages may resonate with some potential participants, research is unfamiliar and abstract to many. Consider instead including a statement about helping improve the way that future treatments are developed. Patients relate more to improving future health than they do to improving future research.
  - If a study is being undertaken as part of a Doctor of Philosophy (PhD) project, consider whether it is necessary for the invitation to highlight this. The benefit may be perceived as solely for the student.
  - Consider highlighting that participants may learn more about their condition by taking part. Some people like to feel that they might learn something by taking part in research.
  - If a financial incentive is to be offered, make the offer clear. Prize draws can be perceived as confusing or misleading.
  - Consider whether it is necessary to mention that the study is international. This can make people feel that their contribution is insignificant. However, some patient communities, such as rare disease communities, may place greater significance on international studies.
  - Avoid referring to patients’ expertise. This can give them a sense of imposter syndrome. Instead, acknowledge the value of their experience.
  - Avoid referring to the core outcome set study as a rare or one-off opportunity. Potential participants may feel such statements are coercive.
4. When explaining the study benefits, consider asking a high-profile patient or a patient organisation to write this section, outlining in one or two sentences why they feel the study is important. Patients want to know if the study is important to other patients and why. It seems to matter less that the researcher feels the study is important. If the researcher themselves has relevant personal experience that they are happy to highlight, this might further illustrate why the research is so important.
5. If you’ve had patients involved in creating your study, say this. For example: “Patients with xxx (health condition) helped develop this study”. This can be reassuring, again reinforcing that other patients feel the study is important and that the researcher values the patient perspective.

**Explaining the study**

1. In a brief sentence explain what potential participants are being asked to do. For example, “You’ll be asked to complete two online surveys, each taking around 20 minutes”. Participants need basic information to work out whether participation is feasible. However, avoid information overload and duplication in invitations—further detail can be provided in the participant information sheet.
2. If you provide bullets about eligibility criteria, make sure it is clear whether participants must meet one or all the criteria.
3. Create a tone that is emotive, enthusiastic, non-coercive and non-demanding, while remaining professional and respectful. Be mindful of anything that could be perceived as suggesting a power imbalance, for example between the researcher and the potential participant, such as “contact my secretary”.

**Keep the invitation simple and understandable**

1. Follow plain language guidance (see example resources below). Including for example:
  - Avoid jargon, acronyms and abbreviations unless the abbreviation is widely known, such as HIV. Remember also that some patients may not remember the medical name of their condition, particularly if the condition was acute and some time ago.
  - Use the active voice. Using pronouns such as ‘you’ or ‘we’ can also help build a relationship with the person.
  - Keep sentences short and simple (maximum 15–20 words). Complex sentences can become confusing and clumsy, which can create mistrust in the research team (people may think ‘if the researchers can’t write a simple sentence how can they run a research study?’).
  - Avoid disclaimers and ensure information about your study’s funding is kept brief. Detailed information on these add to the length of the invitation and disclaimers can create uncertainties and trust issues; people may be anxious about why such statements have been included.
  - Carefully consider all words and phrases in your invitation, particularly those that researchers use every day. For example:
    - The word ‘outcome’ is often misunderstood. If you feel you must talk about outcomes, use a simple example to explain what you mean.
    - Don’t describe what a core outcome set is in your initial invitation. It’s a difficult concept to grasp and can be misunderstood.
    - Avoid using words such as ‘feasibility study’, ‘two rounds’, ‘Delphi’ etc, as they are often misunderstood.
    - Be aware that terms which are commonplace in research might have negative connotations for the public. ‘Principal Investigator’, might lead potential participants to think they will be scrutinised. Words such as ‘critical outcomes’ may have negative associations for patients with experience of critical care situations.
    - Avoid words such as ‘invaluable’. The prefix ‘in’ is often associated with lacking something, so can confuse.

**Amount of information**

1. Keep the invitation very brief (for example, around 200 words in an email or flyer invitation). The public prefer information that is quick and easy to process. They told us that they might not read, or only skim read invitations that are long or textually dense. This is particularly important for people with complex health conditions and carers, who often have many demands on their time and energy.
2. Consider which information is a priority for including at the invitation stage. For example, do you need to tell the participants at the outset which other stakeholders are taking part, or is it more important that they know participation involves completing two online surveys?

**Presenting information**

1. Use bullets and white space to help structure and break up the information. While text boxes can be popular for breaking information up, be aware that these can cause accessibility challenges for people using assistive technology.
2. Consider using images to gain the participant’s attention but make sure the images closely match the messages you wish to convey. For example, a researcher might include a photograph of a person doing meditation to illustrate the well-being aspect of mental health. In an invitation for a study about treatments for depression, this image might imply that only people who have recovered from mental health difficulties are eligible for the study. Guidance is available (see example resources below).
3. If you include a photograph of the lead researcher or the research team, make sure these look professional. Some patients like to see a photograph of the researcher, others don’t, and some find images that look informal to be off-putting.

**Accessibility**

1. To address accessibility challenges, consider a range of methods for disseminating invitations. For example, written letters rather than emails (to address digital literacy), easy read formats for people with learning disabilities, etc. If you suggest participants make contact by telephone, be conscious that some may have hearing difficulties. Guidance is available (see example resources below). If invitations are provided in accessible formats, this will need to be followed through with all other participant-facing study resources.
2. Always check the design and structure of your invitation for accessibility, for example font size, colours etc. Guidance is available (see example resources below).
3. Make accessing the participant information sheet or registering interest easy. A Quick Read (QR) code may be helpful for some, however, not everyone will be familiar with using them and cyber security concerns could be an issue.

**Example resources**

Plain English Campaign—Plain English free guides

Learning and Work Institute—How to prepare clear written materials for a range of readers

Cochrane—Selecting and getting photos

Gov.uk—Making written information easier to understand for people with learning disabilities

Royal National Institute for the Blind—Creating accessible information and communication resources for health and social care (section on Making printed information accessible)

Royal National Institute for the Deaf—How to communicate with someone who is deaf or has hearing loss

COS, core outcome set; PPI, patient and public involvement.

## Discussion

As the first document that potential patient participants in most COS studies will see, the research invitation must be clear, accessible and interesting. The guidance developed in the current study is for COS developers recruiting patient and public participants into COS Delphi studies through remote methods such as email and flyers. It was developed in the UK but may have relevance internationally. We envisage that the guidance will help COS teams consider what to include in their invitations, the amount of information and the kinds of messages that will likely resonate well with patients. The guidance should also help them plan their recruitment approach, particularly given the concerns raised in the GRIS qualitative study about trusting the source of the invitation and worries about clicking on links. Much of the guidance, particularly around the amount of information to present and confusion with terminology, will be relevant to other participant-facing resources in COS studies and aspects of the guidance may be helpful for other health research studies that are recruiting remotely,

Consensus meeting participants included patient research partners, COS developers, a researcher involved in an ethics committee and professionals with expertise in communication, marketing and diversity. As a result of the consensus process, the initial version of the guidance was re-ordered to ensure it mirrored the sequence of the COS invitation development and the dissemination process. The initial version was also restructured to ensure the document was streamlined and easy to use. This guidance can now be used by COS teams; nevertheless, we consider it to be a living document that will likely benefit from being evaluated and refined based on the experience of future COS developers. We also recognise that the guidance will need further refinement in response to wider developments, such as the fear of being scammed becoming more intense with the evolution of artificial intelligence, and the increasing challenge of ‘deep fake’ impersonations.^37^

The consensus meeting participants were also keenly aware that the invitation is only one piece of participant-facing communication in a COS study and that it is futile to improve the invitation but then confuse participants with complex participant information sheets or instructions for Delphi studies. While many of our recommendations are relevant across all such materials, further research is needed to understand the information needs of patients and the public in respect of these other materials, so that COS recruitment and participation are as accessible, understandable and engaging as possible.

This guidance only has the potential to improve COS research if COS developers are aware of it. The international COMET Initiative brings together communities of COS developers and, alongside other COMET resources, will send out this guidance to COS developers when anyone registers a COS study with the Initiative.

### Study strengths and limitations

This study and the recommendations it produced benefited from the expertise of a broad range of stakeholders, including five patient research partners. The study also benefited from being informed by our prior qualitative research with patients and the public using real examples of COS invitations. The guidance was, however, developed with predominantly UK-based participants. It is likely that factors such as cultural differences and variation in accessibility issues (eg, digital literacy) may affect the relevance of the guidance outside the UK context. Evaluation of the guidance outside of the UK would help in understanding such issues. Additionally, most of our participants were aged over 35 and this may have influenced discussions relating to the use of technology, as older consensus meeting participants will likely be less familiar with technology than younger groups.

## Conclusion

COS development is crucial to ensure clinical trials are efficiently designed and patient-centric. Patients are therefore crucial participants in the COS development process, but there are challenges in recruiting them. This guidance aims to help COS teams address these challenges. Through a two-step process, we have sought the views of patients and the public about sampled COS invitations, used the findings of this research to create a list of recommendations and refined these through a consensus process with relevant experts, including patient research partners. While the guidance will benefit from further testing, it provides valuable advice for COS developers about to embark on study recruitment. Through our involvement with the COMET Initiative, we will seek to disseminate the guidance widely.

## Supplementary material

10.1136/bmjopen-2025-111017 online supplemental appendix 1

10.1136/bmjopen-2025-111017 online supplemental appendix 2

10.1136/bmjopen-2025-111017 online supplemental appendix 3

10.1136/bmjopen-2025-111017 online supplemental appendix 4

## Data Availability

No data are available.
