# Supplementary material for: What makes a good research invitation for patient and public participants in core outcome set studies: a UK-based consensus meeting to develop guidance
Source: BMJ Open. 2026 Aug 18;16(8):e111017. doi: 10.1136/bmjopen-2025-111017 (PMC13504522; doi:10.1136/bmjopen-2025-111017)
Supplement: Supplementary file 1 [file bmjopen-2025-111017.reviewer_comments.pdf]

PEER REVIEW HISTORY

BMJ Open publishes all reviews undertaken for accepted manuscripts. Reviewers are asked to complete a checklist review form and are provided with free text boxes to elaborate on their assessment. These free text comments are reproduced below.

ARTICLE DETAILS

Title (Provisional)

What makes a good research invitation for patient and public participants in core outcome set studies: A UK based consensus meeting to develop guidance.

Authors

Barrington, Heather; Craven, Rebecca; Williamson, Paula; Young, Bridget

---

VERSION 1 - REVIEW

**Reviewer**	**1**
**Name**	**Cristall, Nora**
**Affiliation**	**University of Calgary Cumming School of Medicine, Medicine**
**Date**	**01-Jan-2026**
**COI**	**None**

This is an important topic and you have identified practical strategies to assist patients in COS development.

1. Overall comments: This is an important topic, and the authors highlight key issues in patient involvement. There was some confusion in the goals of the study as issues regarding patient involvement in COS selection appeared to be mixed with research in general. It should be clearly stated that the outcomes are for clinical trials as this seems to be the intention. Is there any value in changing the term patient to "people with lived experience" as the participants may no longer be 'patients" or the patient receiving care in the specific example. In this article, likely both terms are appropriate and can be distinguished.
2. There were some noted typo's and extra words. For example, on page three line 60 the word should be removed. On page five there appear to be typo's on line 26, 32, 48-49.
3. On page 3 lines 46-47 the topic of neonatal studies is introduced. Do the authors want to include other clinical populations. Also, in this example the "patient" is the neonate so this should be presented as patients and care providers or guardians.

4. On page four the term ‘trialist’ appears to be vague, can you indicate what is meant by the term or use another word.
5. On page five you indicate you involved an expert in diversity. Can you provide more context to this expertise as this would greatly vary the perspective, world view and ontology of the participant.
6. Table 1 describes the participant demographics, and it is noted that it is a middle and older age population. This should be identified as a shortcoming as in the neonatal example most participants would be <35 years of age. Also, the experience and comfort of younger participants with technology is much different and would provide valuable going forward information. This should be identified as a limitation.
7. Page 5 should include more information on how experts were recruited and their background.
8. Page 13, the authors refer to the patient and public involvement, and they only presented and discussed patient involvement. This should be clarified.

**Reviewer**	**2**
**Name**	**Fackrell, Kathryn**
**Affiliation**	**University of Nottingham**
**Date**	**13-Feb-2026**
**COI**	**None**

This consensus study on developing guidance for invitations to COS studies is well-written and using recognised consensus methodology to identify and agree a final set of key recommendations and guidance for invitations and has clearly reported this process throughout. Given the importance of involving and recruiting patients and public to COS studies to ensure relevant outcomes, I think this study is important and relevant to a wide audience and could apply to many different studies beyond COS.

My only main comment is that it would be really useful if you would provide an example of an invitation using the recommendations that you provide. Just give an idea of how to incorporate the recommendations. Otherwise, I only have some minor comments to correct errors.

Pages 3-4. Patient and public involvement abbreviation has not been defined the first time it was used. Following on from this, the abbreviations for PPI are not used consistently.

I think it would be useful to explain what are public research partners as some people may not know this.

Page 3, Line 60. “patients had been little choice in” Sentence is missing ‘given’

Page 5, Line 32. “When planning the consensus meeting the study team (HB, BY, PW and RC) we aimed for” Need to remove ‘we’ and also add comma after meeting.

VERSION 1 - AUTHOR RESPONSE

Reviewer 1
1. There was some confusion in the goals of the study as issues regarding patient involvement in COS selection appeared to be mixed with research in general.

We feel that we could have been clearer when distinguishing between patient participation and patient involvement in COS studies. We have made some revisions to make the distinction between involvement and participation in COS studies clearer, adding three sentences to paragraph 4 of the introduction to clarify this.

2. It should be clearly stated that the outcomes are for clinical trials as this seems to be the intention.

We have added the words “clinical trials” in the relevant sentence of paragraph 2 and 4 in the introduction.

3. Is there any value in changing the term patient to “people with lived experience” as the participants may no longer be ‘patients” or the patient receiving care in the specific example. In this article, likely both terms are appropriate and can be distinguished.

We would prefer to keep the term ‘patients’, particularly given that public participants in our qualitative study perceived the term ‘people with lived experience’ to be jargonistic.

4. There were some noted typos and extra words. For example, on page three line 60 the word should be removed. On page five there appear to be typos on line 26, 32, 48-49.

I have carefully reviewed the manuscript and corrected several typos and made some minor refinements to the text in addition those noted by the reviewers.

5. On page 3 lines 46-47 the topic of neonatal studies is introduced. Do the authors want to include other clinical populations.

In paragraph 1 of the Introduction, we have clarified that the neonatal example was just for illustration purposes.

6. Also, in this example the “patient” is the neonate so this should be presented as patients and care providers or guardians.

We hope this is now addressed by our response to bullet 3 above.

7. On page four the term ‘trialist’ appears to be vague, can you indicate what is meant by the term or use another word.

We have added an explanation to paragraph 2 of the introduction.

8. On page five you indicate you involved an expert in diversity. Can you provide more context to this expertise as this would greatly vary the perspective, world view and ontology of the participant.

We have added further detail to the sampling and recruitment of participants section of the Methods section to address this.

9. Table 1 describes the participant demographics, and it is noted that it is a middle and older age population. This should be identified as a shortcoming as in the neonatal example most participants

would be <35 years of age. Also, the experience and comfort of younger participants with technology is much different and would provide valuable going forward information. This should be identified as a limitation.

We have clarified that the neonatal study highlighted was just one example from the COS literature. We have also added the issue of age as a limitation in the Article summary - Strengths and limitations bullets and in the 'Study strengths and limitations' section just after the discussion.

10. Page 5 should include more information on how experts were recruited and their background.

We have added further information to the sampling and recruitment of participants section of the Methods section.

11. Page 13, the authors refer to the patient and public involvement, and they only presented and discussed patient involvement. This should be clarified.

We hope that our responses to point 1 address this comment.

Reviewer 2
1. My only main comment is that it would be really useful if you would provide an example of an invitation using the recommendations that you provide. Just give an idea of how to incorporate the recommendations. Otherwise, I only have some minor comments to correct errors.

We thank the reviewer for this helpful suggestion, however we are concerned that by providing such an example readers may defer to thinking 'one size fits all' whereas research invitations should be adapted to specific contexts, populations and study designs. For this reason, we have chosen not to mock up an example invitation as we hope the guidance will be used flexibly by future COS developers.

2. Pages 3-4. Patient and public involvement abbreviation has not been defined the first time it was used.

Definition now provided in the second paragraph of the Introduction.

3. Following on from this, the abbreviations for PPI are not used consistently.

These abbreviations have been amended throughout.

4. I think it would be useful to explain what public research partners are as some people may not know this.

We have added an explanation of this in the second paragraph of the introduction.

5. Page 3, Line 60. "patients had been little choice in" Sentence is missing 'given'.

Word 'given' has been added.

6. Page 5, Line 32. "When planning the consensus meeting the study team (HB, BY, PW and RC) we aimed for" Need to remove 'we' and also add comma after meeting.

Comma added.

VERSION 2 - REVIEW

**Reviewer**	**1**
**Name**	**Cristall, Nora**

Affiliation **University of Calgary Cumming School of Medicine, Medicine**

Date **19-May-2026**

COI

This is an important topic and the authors have identifies a number of key recommendations for core outcome set studies. This paper may be improved with consideration for the following recommendations.

Abstract.

1. Page 2 line 45. In the design section add the word “on or reword the sentence. I notice that the word “on” seems to be missing in a few areas.
2. Page 3 line 8, also the word “on seems to be missing.

   Introduction.

3. The first paragraph could include other examples. The neonatal patient community is very small and the needs are likely unique to this population. The subsequent paragraphs provide a good review of the topic.

Results

4. Is there more information available on the participants. For example, the region of the UK, ethnicity, how the name was put forward to the researchers for recruitment.
5. On page 9 line 35, point 5: Could the authors state this item in more detail. Maybe start with an example statement.
6. Can the authors develop a companion graphic to articulate the recommendations. This would be more suitable for knowledge translation and patient facing material development.

Study Strengths

7. In Line 20 could the authors re word the first sentence so as to not repeat the word expert.

VERSION 2 - AUTHOR RESPONSE

Abstract.

1. Page 2 line 45. In the design section add the word “on or reword the sentence. I notice that the word “on” seems to be missing in a few areas. **We have added the word ‘on’ to this sentence.**

2. Page 3 line 8, also the word “on seems to be missing. **We have added the word ‘upon’ to this sentence.**

Introduction.

3. The first paragraph could include other examples. The neonatal patient community is very small, and the needs are likely unique to this population. The subsequent paragraphs provide a good review of the topic. **We have added other examples.**

Results

4. Is there more information available on the participants. For example, the region of the UK, ethnicity, how the name was put forward to the researchers for recruitment. **We have added further information about the region and how the name of the participants were put forward to this paragraph as requested. Information on ethnicity was in the results section of the article.**

5. On page 9 line 35, point 5: Could the authors state this item in more detail. Maybe start with an example statement. **This suggestion refers to an item in the finalised, consensus driven guidance that has been agreed by the expert panel– we feel we cannot change this agreed guidance.**

6. Can the authors develop a companion graphic to articulate the recommendations. This would be more suitable for knowledge translation and patient facing material development. **Thank you for this suggestion. This would take some time and involve co-production, for which we no longer have the resource.**

Study Strengths

7. In Line 20 could the authors re word the first sentence so as to not repeat the word expert. **The word expert has been changed to stakeholder in this sentence.**
