# Supplementary material for: What makes a good research invitation for patient and public participants in core outcome set studies: a UK-based consensus meeting to develop guidance
Source: BMJ Open. 2026 Aug 18;16(8):e111017. doi: 10.1136/bmjopen-2025-111017 (PMC13504522; doi:10.1136/bmjopen-2025-111017)
Supplement: Supplementary file 2 [file bmjopen-16-8-s001.docx]

**Appendix 1**

**Study Protocol**

**Contents	Page**

1. Proposed start & end date	3
2. Introduction	3
   4.1 Core Outcome Sets (COS)
   4.2 COS study accessibility
   4.3 COS study recruitment messages
   4.4 Empowering patients to take part in COS studies
3. Methods	4
   3.1 Background
       3.1.1 Prior collation of COS study invitations	4
       3.1.2 Overview of studies	5
   3.2 Study 1 - Exploration of the accessibility and engagement of COS	5

invitations

3.2.1 Research question
3.2.2 Objectives
3.2.3 Public/patient focus groups
3.2.4 Analysis
3.2.5 Ethics
3.3 Study 2 - Identification of issues to consider when inviting patients to	9-10

participate in a COS study

3.3.1 Research question
3.3.2 Objectives
3.3.3 Expert consensus conference
3.3.4 Analysis
3.3.5 Ethics

4. Data protection and confidentiality	10
5. Study monitoring	10
6. Dissemination	10
7. References	11
8. List of Appendices	12

**1. Proposed start and end date**

15/05/23 - 15/11/24

**2. Introduction**

***2.1 Core outcome sets***

To help patients, carers, and health professionals make treatment decisions, we need evidence about what works best. Treatments are developed and tested in clinical trials to ensure they work and are safe. To do this, researchers need to examine the effects those treatments have on patients. Researchers do this by measuring an 'outcome'. An outcome in a diarrhoea trial might be how long the symptoms last or how dehydrated the person becomes.

At the moment, different studies looking at treatments for the same condition often measure different outcomes, meaning that we cannot directly compare or combine their results - we would not be comparing like with like. Research needs to be improved so that all studies on a particular health condition use the same outcomes, a 'core outcome set' (COS). A COS refers to the agreed set of outcomes for a particular health condition. If all studies in that health condition measured this COS, we could use the results of all trials to reduce waste and improve the available evidence to support decision-making by patients, carers, and health professionals.

Deciding which outcomes should be core requires considerable discussion. COS studies use 'consensus methods', such as surveys, meetings and discussions to agree on the core outcome set. The patient perspective is considered crucial when agreeing on patient-important outcomes(1). Without this perspective, some COS studies have missed outcomes that have not been identified by clinicians and researchers alone(2). Patients are increasingly being included as key stakeholders in agreeing on core outcome sets(3).

There are greater challenges recruiting patients than retaining them in such studies(4). Fundamental to recruiting patients into COS studies is the invitation to the research. Barrington et al., 2021(4) found that most COS studies invite patients electronically, with very few indicating any form of face-to-face recruitment.

Given recruitment challenges in COS studies, it is important to explore what COS developers communicate to patients in study invitations, and this study will explore this. For patients to engage with the invitation, they need to be able to read and understand it and feel motivated and empowered enough to read beyond the first sentence/subject line. We will examine how patient invitations in COS studies achieve this and will develop guidance for future COS developers.

***2.2 - COS study understanding***

COS studies, like other studies undertaking 'research on research’ (methodological research), can be a challenging area of research for patients to grasp (1, 5). Methodological research has been described as "abstract' as patients are not directly impacted by the research (6). The areas covered may relate to matters that patients are unfamiliar with, such as health economics or data modelling. Patient and public involvement in such areas has been described as requiring a 'steep learning "curve' (7), illustrating the need for more work to support 'patients' understanding of what is involved (6, 8). The challenge of inviting patients to participate in methodological research is that there is insufficient space to provide such extensive information in a short invitation. The focus of the invitation is on providing accessible, understandable and concise information, which, in the field of COS development, is usually provided through text, and rarely in person (4).

In clinical trials, we know that there are challenges with the accessibility of information (9-12). Interventions have been tested in this field to improve the quality and delivery of patient information to improve recruitment. However, these have primarily focussed on participant information sheets and informed consent, and the interventions tested have often failed to improve recruitment or evidence of improvements is uncertain (13). To the authors' knowledge, no previous research has explored how understandable initial invitations to participate in COS studies are for patients.

***2.3 - COS study recruitment messages***

As well as needing to be accessible, the invitations will need to be able to engage patients so that they want to participate. Patients might be unlikely to open an email if the subject header fails to resonate with them. If the email language is unsuccessful in making the patient feel empowered or fails to show how important their perspective is in agreeing a COS, they might choose not to participate. While research invitations must be transparent to avoid patients being manipulated or coerced into research participation, their voices in COS development will not be represented adequately without encouraging and empowering the patients to participate. This risks important outcomes not being prioritised and the potential continuation of research waste, as well as potentially affecting the inclusivity and diversity in participation in COS studies.

To the authors' knowledge, no previous research has explored what messages patients find engaging when being invited to participate in research studies.

***2.4 – Empowering patients to take part in COS studies***

The COS-STAD (standards for COS development) recognise the vital perspective of patients and the public as key stakeholders in COS development(14). Knowledge about outcomes that are important to patients is co-produced in COS studies through the participation of patients and other key stakeholders in consensus-building activities(2). This co-production of knowledge requires patients to feel empowered enough to participate. Being invited to share views on which outcomes should be measured in research studies could feel quite alien to patients(1). Whilst there is increasing emphasis in health care on patient centricity, participation and empowerment(15), being invited into the world of trials methodology research is potentially foreign to patients. Whilst studies have explored how power is communicated in health promotion/medicines information leaflets(16)(17), to the 'authors’ knowledge no studies have explored patient empowerment in patient invitations to research studies, in particular COS studies.

**3 - Methods**

***3.1 - Background***

***3.1.1 - Prior collation of COS study invitations***

We have already collated publicly available COS study invitations from COS developers who have recently completed their studies.

COS studies were sampled via the regularly reviewed and updated COMET Initiative database of COS studies, which includes contact details of chief investigators. As part of COMET’s 2022 database update, COS developers of ongoing COS studies were emailed and asked if they had completed the recruitment of patients into their consensus process and whether invitations were written in English. Those COS studies that had undertaken their patient recruitment for the Delphi aspect of their COS study and were written in English language (for pragmatic reasons) were identified. Additionally, the COMET database was searched to identify published COS studies that had included patients as participants in their Delphi consensus process and had been published since January 2020. It was envisaged that patients will have been recruited more than 3 years ago if the study was published before this date). These more recent COS studies provide an indication of how COS research teams are currently communicating with potential patient participants. Additionally, contacting more recently recruiting studies helps to ensure that study coordinators and patient organisation contacts are still traceable and that any online materials (such as website newsletter articles) are still accessible.

The chief investigator of each of the identified COS studies was emailed, provided with a study information leaflet (advising them that a sample of the invitations collated would be taken to focus groups with patients/the public) and invited to share copies of mass invitational emails/newsletter articles/social media posts/posters/videos/flyers etc that had been used to recruit adults or adult carers into their studies. In some instances, these included posts made on publicly available patient/public social media accounts. The names and contact details of researchers in any of the collected invitations will be removed prior to use in the focus groups. In the final write-up of the focus group study, details that could identify the COS researchers will not be included.

***3.1.2 - Overview of studies***

Two studies will be undertaken to discover and describe what makes a good research invitation for use in COS studies (Figure 1). In Study 1, a purposive sample of the collated COS study invitations (as described in section 5.11) will be examined in focus groups with patients and the public. We aim to explore with the participants how understandable, engaging and empowering they perceive the invitations and their views on other potential approaches to encourage patient participation in COS studies. Then, in Study 2, an expert consensus meeting will be held to discuss findings from Study 1 and from the literature in this area, in order to agree guidance for COS developers on inviting patients into their studies.

***3.2 –*** ***Study 1 - Exploration of the accessibility, empowerment and engagement of COS invitations***

**3.2.1 – Research question**

How engaging, empowering, and understandable are COS study invitations from the perspective of patients and the public?

**3.2.2 – Objectives**

From a patient/public perspective:

- Describe the understandability of the sampled COS study invitations
- Highlight examples of text from COS study invitations that help or hinder patient understanding
- Identify the effectiveness or ineffectiveness of the key persuasive messages used by COS developers
- Reflect on how appealing alternative persuasive messages might be

**3.2.3 - Public/patient focus groups**

Focus groups will be used to explore public and patient views on the understandability of COS study invitations and how engaging and empowering they are. Currently, around 40 COS study invitations have been received. Around six invitations will be taken to the focus groups for discussion. These invitations will be sampled based on criteria such as:

- whether the COS study relates to a predominantly acute or chronic condition (we recognise that where patients have a long-term health condition, for example, in the case of a condition such as diabetes, the researcher’s style of communication and the messages they use could be different than in a more acute condition, such as appendicitis); and
- the method of invitation delivery (we anticipate that different messages will be presented depending on the mode of delivery).

We aim to conduct the focus groups with both members of the public and with patients from community/patient groups. The community/patient groups (non-NHS) will be purposively sampled to include different socio-economic groups, genders, ages and ethnicity. We will access a range of different groups.

Access will be negotiated through the facilitators of the groups we are targeting (either through telephoning or sending written communications such as letters/ emails/text messages - Appendix 1). Further contact will be made if we do not get a response. The researcher will seek to develop rapport and trust so the facilitator feels comfortable enabling access(18), for example, by visiting in person and sharing links with them about the COMET Initiative and the University of Liverpool (communications about core outcome set studies and information about the research team). It is anticipated that up to eight focus groups will be conducted. Of these, around five will be with selected public community groups and around three with non-NHS patient groups.

Once access has been granted, the researcher will ideally seek to attend a regular meeting of the group (physically or virtually) where she can introduce the study and build a rapport with potential participants. If this is not possible or if certain members are not available to attend the meeting, the gatekeeper will be invited to send them an invitation, a participant information sheet, an expression of interest form and a stamped addressed envelope. For anyone not attending the face to face introduction meeting, the initial invitation email/letter introducing the study, participant information sheet, consent form and flyer for this study are provided in Appendix 2a&b -4. An expression of interest form (Appendix 5) will be used for people to register their interest. This includes some basic demographic questions, which will be used to purposively sample participants should more individuals show interest than places available (and potential participants will be made aware of the reason for requesting the information). We will also use this data to describe the characteristics of the study participants and in the interpretation of the study results.

Sampling of participants (if there is a high level of interest) will be purposive for diversity in terms of gender, age, postcode (Index of Multiple Deprivation) and ethnicity (as relevant to the particular group approached, e.g. in an all-male group sampling will be by age, ethnicity and postcode). If the community/patient group facilitator allows, posters advertising the opportunity to participate in the study may be placed in the group setting (Appendix 6). We will offer participants a £15 voucher for attending the meeting to thank them for their participation. The voucher will be suitable for spending in many high street stores. Reasonable travel expenses from the local area will be offered (Appendix 3b). We aim to include up to eight participants in each focus group meeting. However, a reserve list will be held (in case an intended participant cannot attend) should there be a great deal of interest, and potential participants will be informed about this.

Wherever possible, the focus group meeting will be held within the group’s usual meeting place or as close to this as possible to ensure ease of access. Should new Covid regulations/challenges present, or should the group prefer not to meet face to face, online meetings will be held instead, but our preference will be for a face-to-face meeting to facilitate group discussions, rapport building and non-verbal communication. Hybrid meetings will not be attempted as they are known to be alienating for those attending online(19). Should anyone wish to participate but prefers to do so in a one-to-one online interview, we will try to accommodate this. To be eligible to participate in the focus groups/interview, the participants will be aged over 18, be able to speak English and read and have never previously taken part in a COS. Ground rules will be set for the focus group meeting (Appendix 7). If we need to run the focus group online participants will be provided guidance on the secure video conferencing platform (Appendix 8). For the face-to-face focus groups, reasonable travel expenses will be covered and travel booked where required.

Purposively selected participants will be invited to the focus group meeting using their contact details in the expression of interest form (Appendices 9a-9c). If more people are interested than there are places, anyone not selected will be contacted to inform them of this, advising them also that they may still be contacted should a space become available (Appendix 9d). Participants in the focus groups will complete a consent form before participating in the focus group. For participants in the face-to-face meeting, this will be completed at the beginning of the meeting. For any focus groups completed online, participants will be emailed details of the meeting with an embedded consent form that will need to be completed and returned. Intended participants who have not returned their consent form will be contacted prior to any online focus groups to remind them to do so. A link for an online focus group will only be provided once the consent form is completed and returned. For any interviews completed online, consent will be taken verbally. Each item of the consent form will be read to the participant before the interview, and they will be asked whether they consent. This verbal consent process will be audio recorded, and once consent has been obtained, the recording will be stopped and restarted for the interview. The audio recording will be stored as described in section 23, Data Management. A moderator, and where possible, an assistant moderator will facilitate the focus groups. The moderators will take field notes during and after the focus group to document their reflections. The room for the focus group will be set out so that participants can initially spend time alone, privately reading the sampled invitations, considering how understandable and interesting each invitation is. To support the participants in this task, we may provide a fictional example of an advert for a clinical commissioning group event. We plan to use this to illustrate how we want the participants to review the invitations. We will explain to focus group participants that COS research participants receive both an invitation and a participant information sheet that provides further information in addition to the invitation.

As is commonplace in qualitative research we will adapt how we run the focus groups to the needs and preferences of participants and as we learn what approaches work best to address the study aims. We currently plan that each participant will be provided with prompts of things we want them to consider when reading the invitations. We will ask them to consider how understandable, interesting and empowering the invitations are and which aspects of the invitations they did not like, for example, because they found them difficult to understand or because the information was disengaging. We will provide participants with stickers to record their gut reaction to each invitation and with pens, highlighter pens and post-it notes. We will encourage them to use whichever methods suit them to highlight aspects of the invitations that they find particularly easy to understand, interesting or empowering, and any words or phrases they do not like. Paper will be available if they want to write notes.

The participants will be brought together after a refreshment break for a group discussion (topic guide – Appendix 10 – note: Appendix 10 indicates the sorts of topics that will be covered in the focus group, but these will be refined over time). The focus groups will last for up to two and a half hours. The discussion will include the following:

- Identifying preferred invitations and reasons why
- Assessing participants’ perceptions of how understandable, engaging and empowering the invitations were and
- Questions based on potential approaches to recruiting patients informed by areas such as the psychology of persuasion and decision making

The focus group discussions will be audio recorded with the prior consent of the participants, and a professional GDPR accredited transcription service will transcribe the recordings.

**3.2.4 – Analysis**

The focus group data will be transcribed and pseudo-anonymised. Identifying details such as person names will be replaced with ID numbers in transcripts. After transcribing the focus group discussions, we will draw on the constant comparative approach (20) to iteratively analyse and compare the content of the focus group discussions. The field notes taken during and after each discussion will also inform HB's analysis. The analysis will begin after the first focus group when an initial impression of the data will be formed after reading the transcript and field notes. Coding will then be conducted, and a code book will be developed. The codes will then be synthesised into categories based on the developing themes. A comparison of the themes from each focus group will be conducted simultaneously. For example, we will explore variations in the data or we may reconsider our interpretation of earlier themes based on new insights from later focus groups. We anticipate that the software package NVivo will be used to manage the data for the qualitative analysis.

The review of the anonymised transcripts and discussion of the developing analysis will be conducted with the primary and co-supervisors and the public research partner to help inform the analysis.

**3.2.5 – Ethics**

Approval for this study will be sought from the University of Liverpool Health and Life Sciences Committee on Research Ethics (Human participants, tissues and databases). Potential focus group participants will be recruited at a community/patient group meeting or emailed/posted an invitation by the gatekeeper (Appendix 1), along with the participant information sheet (Appendices 3a &b)and the expression of interest form (Appendix 5). At the beginning of the focus group, participants will complete and sign a consent form before participating in the focus group. Electronic versions of the approved consent form will be completed if any focus groups are held electronically and verbal consent in the case of any interviews. The consent form will advise participants that the consensus meeting will be recorded, and that they are free to withdraw from the study at any time up to the point of anonymisation Only participants who are happy to participate in a recorded consensus meeting will be able to participate.

***3.3 –*** ***Study 2 - Identification of issues to consider when inviting patients to participate in a COS study***

**3.3.1 – Research question**

What guidance should be given to COS developers to help them design good COS research invitations?

**3.3.2 – Objectives**

- Discuss a list of key issues to consider when designing a COS study invitation
- Identify any additional issues that should be considered in invitation design
- Agree on a final list of recommendations for COS study invitation design.

**3.3.3 – Expert consensus conference**

We will organise an online Expert Consensus Conference (ECC) to develop guidance for COS developers about issues to consider when inviting patients to participate in a COS study. There is a paucity in the literature on what makes a good research invitation in the field of trials methodology research. The findings from the focus groups and from a narrative review of the literature will be presented to the expert panel. Through discussions and voting we will seek to reach consensus on the aspects of COS invitation design that are the most important for COS developers to consider when designing their research invitations

We are developing a sampling strategy for the ECC. However, the following groups are likely to be represented: patients with experience of participation in COS studies, COS developers, representatives with marketing/communications background and representation from research ethics groups/organisations. Participants will be invited by email and will be made aware that the consensus meeting will be recorded. We will also advise them that by taking part in the consensus meeting they are consenting to participation in the study. The invitation and participant information sheets are provided in Appendices 11 -12b. The previously described Zoom guidance (Appendix 8) will also be provided. The agenda for the ECC is provided in Appendix 13. At the beginning of the consensus meeting a script reminding participants about participation implying consent will be read (Appendix 14).

A list of potential ‘issues to consider when inviting patients into a COS study’ will be drafted prior to the conference, synthesised from the results of the focus group discussions and the literature in this field. The participants will be sent this list in preparation for the consensus meeting. In the meetings, after setting the ground rules (Appendix 15), we plan to begin by again summarising the results of Study 1 and the narrative review of relevant literature. A discussion of each item on the drafted list of ‘issues to consider’ will follow, with anonymised voting to gain consensus on which items should be included in the final guidance. Participants will be encouraged to identify additional issues that they feel are missing; if any are identified, voting will take place on these items too. Consensus will be achieved when at least 70% of the voting participants score between 7 and 9 on the item.

**3.3.4 - Analysis**

A consensus meeting report will be produced, including the scores given to each of the potential recommendations, and a summary of the discussion around each recommendation will be provided to clarify the voting. Once the consensus meeting report has been drafted, it will be sent to the consensus meeting participants for review to ensure it is a fair representation of the discussion.

**3.3.5 – Ethics**

Approval for this study will be sought from the University of Liverpool Health and Life Sciences Committee on Research Ethics (Human participants, tissues and databases). Potential consensus meeting participants will be emailed the participant information sheet and invited to participate. Participants will be advised that attending the consensus meeting implies consent to participating in a video recorded consensus group meeting and that they are free to withdraw from the study at any time up until the point their data is anonymised

4 **Data protection and confidentiality**

These research studies will fully comply with the Data Protection Act of 1998 regarding handling, processing, and storing the study data. All data (including audio recordings, anonymised focus group, interview and consensus meeting transcripts, field notes and participant contact details) will be kept in locked filing cabinets and/or electronically in a secure file store. Audio files will be deleted once these have been transcribed, checked and anonymised, and the transcripts will be held securely (encrypted) before electronic storage or transfer. All data included in the analysis and write-up will be pseudo-anonymised, removing identifiable features, such as place and person names. Any details necessary for interpretation will be included in the analysis, and participants will be advised of this. However, details that may risk a participant's identification will be removed before the findings are reported. This process will be managed by the PhD student (HB) during the course of transcription, transcript checking and write-up. The audio files will be transcribed (intelligent verbatim) as soon as possible following the focus groups using a professional GDPR accredited transcription company. Each transcription will be assigned a code, and only the PhD student (HB) will have access to identifiable data for participants. All identifiable data details will be destroyed at the end of the study.

5 **Study monitoring**

The PhD student (HB) will regularly report to the Principal Investigator (BY) and the Co-investigator (PW) to ensure the research study conforms to the study protocol and relevant University of Liverpool policies and guidance.

6 **Dissemination**

At the end of this research project, the results will be written up as part of a doctoral thesis by the PhD student (HB) and submitted for examination. The findings of this study will be submitted for publication in peer-reviewed journals and presented at conferences. A co-produced plain language summary of the study findings will also be offered to study participants from the various participating community groups and support groups (in the form of an infographic for focus group participants). The findings will be made available to the public more widely by disseminating information on social media and through networks such as the International Patient and Public Involvement Network.

7 **References**

1.	Young B, Bagley H. Including patients in core outcome set development: issues to consider based on three workshops with around 100 international delegates. Research Involvement and Engagement. 2016;2(1).

2.	Williamson P, Altman DG, Bagley H, Barnes KL, Blazeby JM, Brookes ST, et al. The COMET Handbook: version 1.0: BioMed Central; 2017.

3.	Gargon E, Gorst SL, Matvienko-Sikar K, Williamson PR. Choosing important health outcomes for comparative effectiveness research: 6th annual update to a systematic review of core outcome sets for research. PLoS ONE. 2021;16(1):1-12.

4.	Barrington H, Young B, Williamson PR. Patient participation in Delphi surveys to develop core outcome sets: systematic review. BMJ open. 2021;11(9):e051066.

5.	Biggane AM, Williamson PR, Ravaud P, Young B. Participating in core outcome set development via Delphi surveys: qualitative interviews provide pointers to inform guidance. BMJ open. 2019;9(11):e032338.

6.	Al-Janabi H, Coles J, Copping J, Dhanji N, McLoughlin C, Murphy J, et al. Patient and Public Involvement (PPI) in Health Economics Methodology Research: Reflections and Recommendations. Patient. 2021;14(4):421-7.

7.	Stewart DC, Bower, P., Woolfall, K. & Gillies K. Actively Involving Patients/Public with Trials Methodology Research - Report of a Workshop. 2019.

8.	Staniszewska S, Hill EM, Grant R, Grove P, Porter J, Shiri T, et al. Developing a Framework for Public Involvement in Mathematical and Economic Modelling: Bringing New Dynamism to Vaccination Policy Recommendations. The Patient - Patient-Centered Outcomes Research: An Official Journal of the International Academy of Health Preference Research. 2021;14(4):435-45.

9.	O'Sullivan L, Doran P, Sukumar P, Crowley R, McAuliffe E. Readability and understandability of clinical research patient information leaflets and consent forms in Ireland and the UK: A retrospective quantitative analysis. BMJ Open. 2020;10(9).

10.	Terblanche M, Burgess L. Examining the readability of patient-informed consent forms. Dove Medical Press; 2010.

11.	Ménoni V, Lucas N, Leforestier J-F, Doz F, Chatellier G, Jacqz-Aigain E, et al. Readability of the Written Study Information in Pediatric Research in France. PLoS ONE. 2011;6(4):1-6.

12.	Nair SC, Ibrahim H, Askar OS. Comparison of good clinical practice compliance and readability ease of the informed consents between observational and interventional clinical studies in the Emirates. Perspectives in Clinical Research. 2016;7(3):123-7.

13.	Treweek S, Pitkethly ,M, Cook, J, Fraser, C, Mitchell, E, Sullivan, F, Jackson, C, Taskila, TK, Gardner, H. Strategies to improve recruitment to randomised trials. Cochrane Database of Systematic Reviews. 2018;Art. No.: MR000013.(2).

14.	Kirkham JJ, Davis K, Altman DG, Blazeby JM, Mike M, S T, et al. Core Outcome Set-STAndards for Development: The COS-STAD recommendations. PLoS Medicine. 2017;14(11):e1002447-e.

15.	Castro EM, Van Regenmortel T, Vanhaecht K, Sermeus W, Van Hecke A. Patient empowerment, patient participation and patient-centeredness in hospital care: A concept analysis based on a literature review. Patient Education and Counseling. 2016;99(12):1923-39.

16.	van den Berg M, Donyai P. How was patient empowerment portrayed in information leaflets describing the community pharmacy Medicines Use Review service in the UK? Patient Education and Counseling. 2010;80(2):274-6.

17.	Askehave I, Zethsen KK. "Check it out" - The construction of patient empowerment in health promotion leaflets. Utrecht Studies in Language and Communication. 2010(22):105.

18.	Okumus F, Altinay L, Roper A. Gaining access for research: Reflections from Experience. Annals of Tourism Research. 2007;34(1):7-26.

19.	Ellis R, Goodacre T, Mortensen N, Oeppen RS, Brennan PA. The application of Human Factors at Hybrid meetings: facilitating productivity and inclusivity. British Journal of Oral & Maxillofacial Surgery. 2021.

20.	G. GB. The Constant Comparative Method of Qualitative Analysis. Social Problems 1965;12:436-45.

**10. List of Appendices (included as separate documents)**

Appendix 1 – Initial email to community/patient group leader

Appendix 2a - Face-to-face focus group email invitation

Appendix 2b - Online focus group email invitation

Appendix 3a – Focus group participant information sheet

Appendix 3b – Focus group Travel information

Appendix 4 – Focus group consent form

Appendix 5 – Focus group expression of interest form

Appendix 6 – Focus group flyer

Appendix 7 - Focus group ways of working

Appendix 8 – Zoom instructions

Appendix 9a – Invitation to face to face focus group for selected participants

Appendix 9b – Invitation to online focus group for selected participants

Appendix 9c – Invitation to interview for selected participants

Appendix 9d – Not selected for focus group email

Appendix 10 – Focus group topic guide

Appendix 11 – Consensus meeting email invitation

Appendix 12a – Consensus meeting – Professionals participant information sheet

Appendix 12b – Consensus meeting – Public research partners participant information sheet

Appendix 13 – Consensus meeting agenda

Appendix 14 – Consensus meeting Ground rules slides
