# Supplementary material for: What makes a good research invitation for patient and public participants in core outcome set studies: a UK-based consensus meeting to develop guidance
Source: BMJ Open. 2026 Aug 18;16(8):e111017. doi: 10.1136/bmjopen-2025-111017 (PMC13504522; doi:10.1136/bmjopen-2025-111017)
Supplement: Supplementary file 3 [file bmjopen-16-8-s002.docx]

**Appendix 2**

**Draft recommendations for creating core outcome set study invitations**

This guidance is for core outcome set teams. Our main aim in these recommendations is to enhance the initial invitations or communications sent to potential participants in core outcome set studies, although we do note some implications for the information sheets/materials that research participants receive. The recommendations have been developed through a two-stage process: (1) focus groups with a diverse range of patients and the public from the UK who explored examples of previous core outcome set invitations (2) a consensus meeting attended by mostly UK-based participants with experience in core outcome set development, patient and public involvement, science communication, diversity and ethics committees. It should be noted that language and cultural differences alongside logistical challenges outside the UK may mean the recommendations need adjusting for other geographical locations.

When we refer to the public in this guidance we are talking about patients, carers and general members of the public who may be invited to participate in a core outcome set study.

**Reaching your audience**

1. **Provide alternative methods for the participant to verify the invitation** e.g. a telephone number for contacting the team for reassurance**.** The public are concerned about online safety. They told us that receiving an unsolicited email invitation from a stranger can be concerning, especially if they are asked to ‘click on a link’ for further information or register their interest. Even including logos from trusted organisations often fail to reassure as the public are aware that these can be easily copied.
2. **If you suggest participants make contact by telephone, be conscious that some may have hearing difficulties**.
3. **Consider alternative ways of disseminating your invitation or recruiting participants**. The public are suspicious of e-invitations and told us they have greater confidence in invitations if these are provided in trusted spaces, e.g. a poster on the doctor’s clinic wall, a newsletter on a patient organisation website and face-to-face invitations. Receiving a signed letter through the post might also address some of the concerns raised about cyber security.

**Engaging potential participants**

4. **Think about how meaningful study titles are to potential participants.** If you choose to include a study title on the invitation, it should be simple and immediately resonate with them. Avoid using an acronym in the study title – it requires decoding before considering whether the study is relevant. The use of upper- and lower-case letters or altered spelling of familiar words in acronyms further bewilders the public.
5. **Create interest in the study and maintain this so people read to the end of the invitation.**
   a. Use questions such as “Do you have xxxx (condition), or are you are carer for someone with this condition? Would you like to help improve the way xxxx treatments are developed”?
   b. Get to the point of the invitation quickly and use language that demonstrates that you (the research team) are enthusiastic about the core outcome set study.
   c. Ensure there is a clear reason for people to take part and emphasise the value of the patient/public perspective.
   d. Describe the study in such a way that it feels easy to take part in.
7. **Think about what your audience will find appealing or off putting**
   a. **Avoid saying “help us improve future research”**. Whilst altruistic messages resonate well with potential participants, research is unfamiliar and abstract to many. Consider instead a statement about helping improve the way that future xxxx treatments are developed for people with the condition. (Patients relate more to improving future health than they do to improving future research).
   b. **Avoid highlighting in the invitation that the study being undertaken for the completion of a student’s PhD** – the benefit may be perceived as solely for the student.
   c. **Consider emphasising the potential educational benefits to participants if they take part**. Some people like to feel that they will learn something by taking part in research.
   d. **If a financial incentive is to be offered, make the offer clear – prize draws can be confusing or misleading.**
   e. **Avoid mentioning that the study is international**, this can make people feel that their contribution is insignificant.
   f. **Avoid referring to patients’ expertise**, this can give them a sense of imposter syndrome, instead recognise their experience.
   g. **Avoid referring to the core outcome set study as a rare/one-off opportunity** – potential participants can find such statements to be overly coercive.
8. **Use pronouns such as ‘you’ or ‘we’ to help build a relationship with the person**.
9. **When explaining the study benefits, consider asking a high-profile patient or a patient organisation to write this section, outlining in one or two sentences why they feel the study is important.** Patients want to know if the study is important to other patients and why. It seems to matter less that the researcher feels the study is important. If the researcher themselves has relevant personal experience that they would feel happy to highlight – this might further illustrate why the research is so important.
10. **If you’ve had patients involved in designing the study, say this**, for example: “Patients with xxx (health condition) helped us design the study”, this can be reassuring, again reinforcing that other patients feel the study is important and that the researcher values the patient perspective.
11. **Consider using AI to create engaging invitations for the public.**

**Explaining the study**

12. **Tell the participant what you are asking them to do, how much time it will take, what the benefits might be and how they can take part**. Again, avoid information overload. Participants receive additional information in the participant information sheet so minimise duplication. Be especially clear about how people take part - potential participants need to work out whether participation is feasible for them. For example, confirm if the study is online, how many surveys there will be, whether hard copies are available, when they will receive the survey(s) and how long they will take to complete.
13. **If you provide bullets about eligibility criteria, make sure it is clear whether participants must meet one or all the bulleted criteria.**
14. **Create an emotive, enthusiastic, non-demanding tone, but remain professional and respectful.** Be mindful of anything that could be considered hierarchical, or suggestive of a power imbalance.
15. **Consider the potential burden of your study, minimising the amount of time needed to take part.** Potential participants are very influenced by the time commitment needed when deciding whether to take part

**Keep it simple and understandable**

16. **Keep sentences short and simple (maximum 15-20 words).** Complex sentences can become clumsy, which can create mistrust in the research team (the public may think ‘if the researchers can’t write a simple sentence how can they run a research study?’).
17. **Avoid including detailed funder information or disclaimers.** These add to the length of the invitation. Disclaimers can also create uncertainties and trust issues; people may be anxious why they are there.
18. **Avoid jargon and acronyms.** Even if you think a person might be familiar with them, they may not be. The participant may be new to the condition. When you use a term, consider whether the general public use that term. Recognise the challenges of health literacy. Remember also that some patients, particularly if the condition was acute and some time ago, may not remember the name of their condition. [Guidance](https://www.plainenglish.co.uk/free-guides.html) on writing in plain language is available.
19. **Carefully consider all words and phrases in your invitation, particularly those that researchers use every day.**
    a. The word ‘outcome’ is often misunderstood. If you feel you must talk about outcomes, use a simple example to explain what you mean.
    b. Don’t describe what a core outcome set is in your brief invitation, it’s a difficult concept to grasp and can be misunderstood.
    c. Avoid using words such as ‘feasibility study’ ‘two rounds’ ‘Delphi’ etc as they are often misunderstood.
    d. Be aware of potential incongruences and sensitivities associated with terms such as ‘Principal Investigator’, which might carry connotations that participants will be scrutinised. Words such as ‘critical outcomes’ may be interpreted negatively by patients familiar with critical care situations.
    e. Avoid words such as ‘invaluable’- the prefix ‘in’ is often associated with lacking something, so can confuse.
20. **Consider using AI to assist in creating simple invitations for the public.**

**Amount of information**

21. **Keep the invitation very brief (for example, about 100 – 120 words for a flyer or email invite)**. The public prefer information that is quick and easy to process. They told us that they might not read, or only skim read invitations that are too long or textually dense.
22. **Recognise that patients and the public have busy lives, particularly if they have a health condition or are a carer**. For example, if they are in a busy waiting room environment with children, they may only have a minute or two to read a poster invitation.
23. **In your invitation, when you direct participants to further study information, make it clear that the information is brief and keep the participant information sheet to one page wherever possible.** If the public think they have to read a further detailed participant information sheet they may be put off reading it and participating
24. **Provide the information you send to people in digestible chunks or segments to help reduce the cognitive load.** For example, a brief participant information sheet could be supplemented by a simple video description of what a core outcome set is.
25. **Consider whether the information you are giving is really necessary at the invitation stage.** For example, do you need to tell the participants at the outset that other stakeholders will take part, or is it more important that they know participation involves completing two online surveys?

**Presenting information**

26. **Use bullets and white space to help structure and break up the information**. [Guidance](https://learningandwork.org.uk/resources/research-and-reports/readability-how-to-produce-clear-written-materials-for-a-range-of-readers/) is available. Whilst text boxes can be popular for breaking information up, be aware that they can cause accessibility challenges for people using assistive technology.
27. **Consider using images to gain the participant’s attention but be aware of any potential incongruences in the images you plan to use**. [Guidance](https://training.cochrane.org/sites/training.cochrane.org/files/public/uploads/Images%20Checklist%20for%20Evidence%20Dissemination%20-%20Final,%20version%201.0_1.pdf) is available.
28. **If you include a photograph of the lead researcher or the research team, make sure these look professional.** Some patients like to see a photograph of the researcher, others don’t, and some find images that look informal to be off putting.

**Accessibility**

29. **Always check the design and structure of your invitation for accessibility, for example font size, colours etc.** [Guidance](https://www.rnib.org.uk/living-with-sight-loss/independent-living/accessible-nhs-and-social-care-information/creating-accessible-information-and-communication-resources-for-health-and-social-care/) is available.
30. **Aim to provide translated versions of your invitation.** Use backward-forward translation to ensure information accuracy. Issues relating to the translation of core outcome set study materials have previously been [discussed](https://trialsjournal.biomedcentral.com/articles/10.1186/s13063-021-05338-x).
31. **Consider offering hard copy surveys to address digital literacy and providing easy read formats for people with learning disabilities.** [Guidance is available](https://www.gov.uk/government/publications/making-written-information-easier-to-understand-for-people-with-learning-disabilities-guidance-for-people-who-commission-or-produce-easy-read-information-revised-edition-2010).
32. **Make accessing the participant information sheet or registering interest easy.** A QR code may be helpful for some, however, recognise that not everyone will be familiar with using them and that cyber security concerns could be an issue.

**Data safety**

33. **Where invitations are being addressed directly to participants, make it clear how you came to have their name.**
34. **Provide clear reassurance on data management.** Statements about ‘anonymity’ appear contradictory to participants who are being emailed the survey using their name. Further reassurance may be needed.

**Producing and user testing your invitation**

35. **Co-produce your study with patients and the public.**
36. **User-test or pilot your study invitation with your target audience** (ensuring they are a diverse group and not research aware patient research partners). Do this even if patient research partners have been involved in the design of your study, as patient research partners are often familiar with medical or research terms.
