# Supplementary material for: What makes a good research invitation for patient and public participants in core outcome set studies: a UK-based consensus meeting to develop guidance
Source: BMJ Open. 2026 Aug 18;16(8):e111017. doi: 10.1136/bmjopen-2025-111017 (PMC13504522; doi:10.1136/bmjopen-2025-111017)
Supplement: Supplementary file 4 [file bmjopen-16-8-s003.docx]

**Appendix 3 - Consensus meeting facilitator notes**

- Welcome - Explain that on the call we have 13 <amend as appropriate> participants including patient research partners, ethicists, core outcome set developers and experts in marketing, communication and inclusive research. Everyone has been invited because they have expertise, either from lived experience or professional experience. Emphasis that their contributions will be incredibly valuable. SG will be our technology person today, dealing with any Zoom queries and voting etc. HB and RC will be presenting. PW, BY and NH will be observing.
- Introductions.
  - Ask each participant to very briefly say their name and the role that they have.
  - Ask each observer / presenter / technological support team member to briefly introduce themselves (their usual role).
- Advise participants - Before introducing the aim of our study today, you will be aware that we will be talking about research invitations used in core outcome set studies. To make sure we all understand what core outcome set studies are HB will begin by providing a very brief reminder of what they are, how they are developed and why patient participation is so important.
- **Introduce HB – COS presentation**
- Provide a reminder of the study’s aim - We want to ensure the best possible research invitations are used when recruiting patients into core outcome set (COS) studies, so we aim to produce guidance for COS developers on what makes a good research invitation. By ‘best possible’ we mean understandable, engaging, non-coercive invitations. When we talk about COS developers, we are assuming that the study’s public research partners are part of the COS development team so the recommendations need to make sense to public research partners who may be new to their role and may use the guidance when involved in designing invitations to be sent to participants.
- Explain that the study team have previously explored a sample of COS study invitations with patients and the public to determine how understandable and engaging they are. Today the team will present a summary of this work and an overview of the draft recommendations (which you will already have received by email).
- Explain that we will then move to discussing each of the draft recommendations, asking about whether the recommendation makes sense and whether they have any comments or concerns about it. We will discuss whether further research is needed to support the recommendation. We will then decide whether we feel the recommendation should be included in the final list of recommendations. Where appropriate we will vote on the recommendation. If 70% agree with its inclusion (9 people if 13 attend) it will be included in the final recommendations. It is OK if consensus cannot be reached, for some of the recommendations. If you do not understand a point being made during the discussion please let me know so I can clarify things, you may find that others in the room have not understood either. As per your pre-meeting information, this discussion will be recorded for note taking purposes only. Attending the consensus meeting implies consent to participation. As people may use examples from their own experience when discussing a recommendation, it is vital that all participants respect confidentiality. Nothing said in the meeting should be repeated outside of the meeting.
- Show slide with the agenda explaining - We have regular breaks in the agenda but if you feel that you need to step away from the meeting at any point please do so. If you do though, it would be helpful if you can let us know in the chat feature that you are stepping away. We need to keep to time so we will start promptly after each break.
- Show ground rules slide – here are the ground rules for this study.
- Check verbal assent to video record the discussion using the following wording “Attending this consensus meeting implies consent to participating in an audio recorded consensus group meeting”.
- Please explain that while Zoom automatically creates both a video and an audio recording, only the audio recording will be used for note-taking. The video recording will be deleted immediately after the meeting. The recording will only be used by HB and BY when reviewing the draft guidance. The recording will not be used for any other purpose.
- SG will now tell us about how we will use Zoom today (HB to check with SG will this include hand raising / chat function and voting).
- **Introduce SG - Zoom explanation**
- General note: Please ensure all attendees have a voice. There are allocated time slots for each recommendation on the agenda (approximately xx minutes <number of minutes will be updated once the recommendations are finalised> for each recommendation).
- **Introduce HB and RC – GRIS focus group study findings.**
- Please read out each recommendation in turn using the slides to scroll through the recommendations and get feedback before moving on to the next. Asking:
  - Is the recommendation clear enough? Any wording changes needed to clarify anything?
  - Do you have any initial comments or concerns about this recommendation? (For example, are there any practical or ethical issues that need to be considered in relation to the recommendation?
  - Do we need more evidence to support the recommendation? If so, what kind of evidence?
  - If everyone is happy with the original (or any revised) wording of the recommendation in terms of clarity, ask if anyone wants to argue against including the recommendation in the guidance. If not, then the recommendation will be included. If anyone disagrees then discuss the recommendation exploring views for and against including it. Then vote on the recommendation.
- Once all the draft recommendations have been discussed, ask the participants, based on the study findings presented by HB and RC are there any recommendations that they haven’t made that participants feel we should have made? If so, what are they, and then get the group to discuss the newly proposed recommendation(s) based on the questions above.
- If there is time to discuss, explain how the guidance will be disseminated (via a published article that will be available on the COMET website, also ultimately in the COMET Initiative’s handbook for developing COS). Did they have any thoughts on other ways of disseminating the top tips / raising awareness of them and making them accessible?

Thank participants for their contributions. With each participant’s permission, we will acknowledge meeting participants in any publications from this study, listing them also in the PhD thesis. Ask if they are willing for HB to contact them after the meeting to check they are happy to be acknowledge by name, to ask if they would like to see a copy of the final report and to clarify anything she needs further information on? Ask that they please keep the results of today’s meeting confidential until the team publishes the findings of the study.
