# Supplementary material for: What makes a good research invitation for patient and public participants in core outcome set studies: a UK-based consensus meeting to develop guidance
Source: BMJ Open. 2026 Aug 18;16(8):e111017. doi: 10.1136/bmjopen-2025-111017 (PMC13504522; doi:10.1136/bmjopen-2025-111017)
Supplement: Supplementary file 5 [file bmjopen-16-8-s004.docx]

**Appendix 4 - Summary of discussions and voting in consensus meeting**

| **Recommendation category** | **Recommendation number** | **Recommendation** | **Discussion points** | **Agreed action** | **Voting took place** | **Recommendation In/out** |
| --- | --- | --- | --- | --- | --- | --- |
| Reaching your audience | 1 | Provide alternative methods for the participant to verify the invitation e.g. a telephone number for contacting the team for reassurance. The public are concerned about online safety. They told us that receiving an unsolicited email invitation from a stranger can be concerning, especially if they are asked to ‘click on a link’ for further information or register their interest. Even including logos from trusted organisations often fail to reassure as the public are aware that these can be easily copied. | Potential participants may be equally suspicious of calling someone eg. premium phone numbers. There may be a range of ways the person could verify the invitation so remove the example. It is also unclear what 'alternative methods' refers to. | Change wording to highlight alternative methods removing the example of the telephone number. Clarify what 'alternative methods' means. | All agreed to action, no voting | In |
| Reaching your audience | 2 | If you suggest participants make contact by telephone, be conscious that some may have hearing difficulties. | Concerns that this fits better with the accessibility topic and could be merged but important to include | Move and merge within the accessibility section but highlight hearing issues and dyslexia as potential examples of accessibility issues | Voted, n = 13 (Only 23% voted to include, 77% voted to move and merge). | In |
| Reaching your audience | 3 | Consider alternative ways of disseminating your invitation or recruiting participants. The public are suspicious of e-invitations and told us they have greater confidence in invitations if these are provided in trusted spaces, e.g. a poster on the doctor’s clinic wall, a newsletter on a patient organisation website and face-to-face invitations. Receiving a signed letter through the post might also address some of the concerns raised about cyber security. | Dissemination of the invitation comes before verification so this should be recommendation 1. The alternative approaches to dissemination may have ethical consequences so this should be specified. Alternative sources could include settings such as mother and toddler groups. 1 is part of 3 and it’s all about developing credibility and trust. To increase participation of people from more diverse backgrounds it's better if the invitation comes from someone like them or someone they trust, emphasise the importance of potentially coming from patient organisations rather than the research team whom they don't know. | Make this recommendation number 1 and adjust wording to: Consider how you are going to disseminate and the implications that this may have on trust and make sure you have the appropriate ethical approval for how you are going to recruit. Ensure that the revised recommendation also includes places like community groups and trusted organisations such as patient organisations - we mentioned patient organisation newsletters previously but emails from them might also be more trusted. | All agreed to action, no voting | In |
| Engaging potential participants | 4 | Think about how meaningful study titles are to potential participants. If you choose to include a study title on the invitation, it should be simple and immediately resonate with them. Avoid using an acronym in the study title – it requires decoding before considering whether the study is relevant. The use of upper- and lower-case letters or altered spelling of familiar words in acronyms further bewilders the public. | Agreed, the title needs to be simple and say what it is about. This recommendation is important but may be hard for researchers to hear - so keep in. | No changes | No changes, no voting | In |
| Engaging potential participants | 5 | Create interest in the study and maintain this so people read to the end of the invitation. **a.**       Use questions such as “Do you have xxxx (condition), or are you are carer for someone with this condition? Would you like to help improve the way xxxx treatments are developed”? **b.**       Get to the point of the invitation quickly and use language that demonstrates that you (the research team) are enthusiastic about the core outcome set study. **c.**       Ensure there is a clear reason for people to take part and emphasise the value of the patient/public perspective. **d.**       Describe the study in such a way that it feels easy to take part in. | Delphi studies are not easy to participate in - it is important to strike a balance between providing clear and transparent information whilst ensuring the invitation looks engaging. Consider removing D, it may not add anything. | Remove D | All agreed to action, no voting | In but remove D. |
| Engaging potential participants | 7 | 7.       Think about what your audience will find appealing or off-putting **a.** Avoid saying “help us improve future research”. Whilst altruistic messages resonate well with potential participants, research is unfamiliar and abstract to many. Consider instead a statement about helping improve the way that future xxxx treatments are developed for people with the condition. (Patients relate more to improving future health than they do to improving future research). **b.** Avoid highlighting in the invitation that the study being undertaken for the completion of a student’s PhD – the benefit may be perceived as solely for the student. **c.** Consider emphasising the potential educational benefits to participants if they take part. Some people like to feel that they will learn something by taking part in research. **d.** If a financial incentive is to be offered, make the offer clear – prize draws can be confusing or misleading. **e.** Avoid mentioning that the study is international, this can make people feel that their contribution is insignificant. **f.** Avoid referring to patients’ expertise, this can give them a sense of imposter syndrome, instead recognise their experience. **g.** Avoid referring to the core outcome set study as a rare/one-off opportunity – potential participants can find such statements to be overly coercive. | C. The term 'educational benefit' may be too grand, instead refer to potential learning (although learning cannot be guaranteed). D. International Rare disease communities may want the statement to say that the study is international so in some situations it may be appropriate to include. F. Don't say avoid, maybe say consider. | 7c. Reword educational benefit to learning. 7e. Reword emphasising that the COS developer should consider whether it is necessary to mention that the study is international. | All agreed to action, no voting | In |
| Engaging potential participants | 8 | Use pronouns such as ‘you’ or ‘we’ to help build a relationship with the person. | This is an example of writing in plain language as it refers to the active voice. Recommendations 16 and 18 are also components of writing in plain language, yet there is no recommendation about using plain language. Suggest bundling the plain language recommendations together into one and maybe having the wording in recommendation 8 as an example) | Bundle with other plain language recommendations (maybe have this as an example) | All agreed to action, no voting | In but only as an example |
| Engaging potential participants | 9 | When explaining the study benefits, consider asking a high-profile patient or a patient organisation to write this section, outlining in one or two sentences why they feel the study is important. Patients want to know if the study is important to other patients and why. It seems to matter less that the researcher feels the study is important. If the researcher themselves has relevant personal experience that they would feel happy to highlight – this might further illustrate why the research is so important. | This is about credibility and trust again | No changes | No changes needed, no voting | In |
| Engaging potential participants | 10 | If you’ve had patients involved in designing the study, say this, for example: “Patients with xxx (health condition) helped us design the study”, this can be reassuring, again reinforcing that other patients feel the study is important and that the researcher values the patient perspective. | Is 'design' the study open enough rather than 'create' the study - create might be simpler. Adds credence and support. 'Helped us' terminology discussed, whilst there were concerns that it may not imply equal partnership, the term co-produced was felt difficult to understand. Initial concern that this recommendation would increase the content of the invitation, however when the popularity of this statement amongst focus group participants was explained consensus meeting participants agreed that it should be included. | Change 'design' to created. | All agreed to action, no voting | In |
| Engaging potential participants | 11 | Consider using AI to create engaging invitations for the public. | This recommendation was not considered helpful as AI is broad and constantly evolving and the recommendation does not explain the ways of using AI. Advised the guidance could equally have recommended that the COS developer refers to someone with public engagement skills. | Remove and merge with co-production referring to the use of tools or other available professionals. | Voted n = 12 (Only 8% voted to include) | Out (merge with co-production) |
| Explaining the study | 12 | Tell the participant what you are asking them to do, how much time it will take, what the benefits might be and how they can take part. Again, avoid information overload. Participants receive additional information in the participant information sheet so minimise duplication. Be especially clear about how people take part - potential participants need to work out whether participation is feasible for them. For example, confirm if the study is online, how many surveys there will be, whether hard copies are available, when they will receive the survey(s) and how long they will take to complete. | The key is the time involved and whether its online or in person might be most important. Reduce what we include and say something briefer and more succinct so that not too much is included in the invitation (concerns about the length of the invitation). | Reword so less for COS developer to include as more detail should be in the PIS. In a brief sentence explain what they are being asked to do: E.g. Take part in two online surveys, that should take around 20 minutes each to complete. | All agreed to action, no voting | In |
| Explaining the study | 13 | If you provide bullets about eligibility criteria, make sure it is clear whether participants must meet one or all the bulleted criteria. | Agreement - no concerns voiced | No changes | No changes, no voting | In |
| Explaining the study | 14 | Create an emotive, enthusiastic, non-demanding tone, but remain professional and respectful. Be mindful of anything that could be considered hierarchical, or suggestive of a power imbalance. | Discussion around whether ethics may see enthusiasm as coercion, however, it was confirmed that enthusiasm is acceptable, it is just unacceptable to exaggerate, such as "this is the best thing ever.' Adjust the wording to be clear that enthusiasm should be non-coercive. The final sentence did not make it clear who might consider wording to be hierarchical. | Change to reflect that this is non-coercive and adjust the statement about hierarchical to improve clarity. | All agreed to action, no voting | In |
| Explaining the study | 15 | Consider the potential burden of your study, minimising the amount of time needed to take part. Potential participants are very influenced by the time commitment needed when deciding whether to take part | Beyond the scope of the guidance as to do with study design. | Remove as beyond scope. | Voted, n = 12, (Only 33% voted to include). | Out |
| Keep it simple and understandable | 16 | Keep sentences short and simple (maximum 15-20 words). Complex sentences can become clumsy, which can create mistrust in the research team (the public may think ‘if the researchers can’t write a simple sentence how can they run a research study?’). | This is all part of plain language writing - bundle plain language recommendations together. Complex sentences are not just clumsy, they are confusing - emphasise this. | Bundle with other plain language recommendations and include reference to complex sentences being confusing | All agreed to action, no voting | In but include in a plain language section |
| Keep it simple and understandable | 17 | Avoid including detailed funder information or disclaimers. These add to the length of the invitation. Disclaimers can also create uncertainties and trust issues; people may be anxious why they are there. | Patients might want to know if a well-known funder has funded the study. Could be bundled with plain language. | Adjust wording to emphasise any funder information should be brief. Include within the plain language section | All agreed to action, no voting | In, include in a plain language section |
| Keep it simple and understandable | 18 | Avoid jargon and acronyms. Even if you think a person might be familiar with them, they may not be. The participant may be new to the condition. When you use a term, consider whether the public use that term. Recognise the challenges of health literacy. Remember also that some patients, particularly if the condition was acute and some time ago, may not remember the name of their condition. Guidance on writing in plain language is available. | It was pointed out that the issue with acronyms may be more context-dependent, for example, spelling out human immunodeficiency virus is wordier than HIV, an acronym widely recognised by the public. This recommendation could also come under plain language. | Adjust wording to highlight example of commonly used acronym that does not require spelling out such as HIV. Bundle with plain language | All agreed to action, no voting | In but bundle under plain language |
| Keep it simple and understandable | 19 | Carefully consider all words and phrases in your invitation, particularly those that researchers use every day. **a**. The word ‘outcome’ is often misunderstood. If you feel you must talk about outcomes, use a simple example to explain what you mean. **b**. Don’t describe what a core outcome set is in your brief invitation, it’s a difficult concept to grasp and can be misunderstood. **c**. Avoid using words such as ‘feasibility study’ ‘two rounds’ ‘Delphi’ etc as they are often misunderstood. **d**. Be aware of potential incongruences and sensitivities associated with terms such as ‘Principal Investigator’, which might carry connotations that participants will be scrutinised. Words such as ‘critical outcomes’ may be interpreted negatively by patients familiar with critical care situations. **e**. Avoid words such as ‘invaluable’- the prefix ‘in’ is often associated with lacking something, so can confuse. | This is not a stand-alone recommendation and could be merged with the plain language recommendations. | Merge with the plain language recommendations | All agreed to action, no voting | In but merge in plain language section |
| Keep it simple and understandable | 20 | Consider using AI to assist in creating simple invitations for the public. | Remove for the same reasons as recommendation 11 | Remove | All agreed to action, no voting | Out |
| Amount of information | 21 | Keep the invitation very brief (for example, about 100 – 120 words for a flyer or email invite). The public prefer information that is quick and easy to process. They told us that they might not read, or only skim-read invitations that are too long or textually dense. | If we are going to include this as a recommendation, we need to make sure the content suggested in the recommendations can fit within 100 - 120 words. The number of words is critical here. | In but tweaking for the amount of words needed to include the other recommendations. | All agreed to action, no voting | In but amend once approximation of word length established after following the finalised recommendations. |
|  | 22 | Recognise that patients and the public have busy lives, particularly if they have a health condition or are a carer. For example, if they are in a busy waiting room environment with children, they may only have a minute or two to read a poster invitation. | What would enacting this recommendation look like that wasn't enacted elsewhere? HB suggested that because this was highlighted as a participant it maybe just needs adding as an example to 21. We could include as an example with 21. | Add as a brief example to 21 | All agreed to action, no voting | Out but keep as an example for 21. |
|  | 23 | In your invitation, when you direct participants to further study information, make it clear that the information is brief and keep the participant information sheet to one page wherever possible. If the public think they must read a further detailed participant information sheet they may be put off reading it and participating | Depends on the paper size. Some had seen 2 pages. This is moving into the realms of participant information - the study is about the invitation, so this is out of scope. | Remove | Voted, n = 10, (Only 10% voted to include). | Out |
|  | 24 | Provide the information you send to people in digestible chunks or segments to help reduce the cognitive load. For example, a brief participant information sheet could be supplemented by a simple video description of what a core outcome set is. | Beyond the scope as to do with participant information sheets. | Remove | Voted, n=8 (Only 25% voted to include) | Out |
|  | 25 | Consider whether the information you are giving is really necessary at the invitation stage. For example, do you need to tell the participants at the outset that other stakeholders will take part, or is it more important that they know participation involves completing two online surveys? | Good as refers to what the purpose of the invitation is. | No changes | No changes, no voting | In |
| Presenting information | 26 | Use bullets and white space to help structure and break up the information. Guidance is available. Whilst text boxes can be popular for breaking information up, be aware that they can cause accessibility challenges for people using assistive technology. | No comments | No changes | No changes, no voting | In |
| Presenting information | 27 | Consider using images to gain the participant’s attention but be aware of any potential incongruences in the images you plan to use. Guidance is available. | Is incongruences the right word - maybe mismatch, may need an example. Lots of points in this guidance about referring to other guidance- are these links going in a footer or appendix too? | Keep in but adjust the wording and potentially an example. Consider where links to other guidance should go. | All agreed to action, no voting | In |
| Presenting information | 28 | If you include a photograph of the lead researcher or the research team, make sure these look professional. Some patients like to see a photograph of the researcher, others don’t, and some find images that look informal to be off putting. | No comments | No changes | No changes, no voting | In |
| Accessibility | 29 | Always check the design and structure of your invitation for accessibility, for example font size, colours etc. Guidance is available. | Merge recommendation 2 into this. | Merge recommendation 2 into this. | All agreed to actions, no voting | In but merge recommendation 2 into this. |
| Accessibility | 30 | Aim to provide translated versions of your invitation. Use backward-forward translation to ensure information accuracy. Issues relating to the translation of core outcome set study materials have previously been discussed. | Whilst this is an aspiration, where does the funding come from, because all participant-facing resources used in the study would need translating? Also, there are so many languages how do you decide which one(s)? If the invitation is going to be specifically for people who speak a particular language, ethics would have mandated that the materials are available in that language, so this does not need to be specifically stated. Because this is a complete study design issue it is beyond scope. One participant had seen examples of where only the study invitation was translated, and all other participant-facing resources in one language. | Remove | Voted, n = 11 (Only 46% voted to include). | Out |
| Accessibility | 31 | Consider offering hard copy surveys to address digital literacy and providing easy read formats for people with learning disabilities. Guidance is available. | This should read hard copy invitations not surveys (change the wording). This needs to be followed through not just for the invitation. | Change the word surveys to invitations, this will need to say something about following through to other documents. | All agreed to actions, no voting | In |
| Accessibility | 32 | Make accessing the participant information sheet or registering interest easy. A QR code may be helpful for some, however, recognise that not everyone will be familiar with using them and that cyber security concerns could be an issue. | No comments | No changes | No changes, no voting | In |
| Data safety | 33 | Where invitations are being addressed directly to participants, make it clear how you came to have their name. | Maybe merge with recommendation around building trust. If directly to participants and it hasn't come from someone that has your address you would have to include this for GDPR. So has to be in. | Consider merging with recommendation about building trust | All agreed to actions, no voting | In |
| Data safety | 34 | Provide clear reassurance on data management. Statements about ‘anonymity’ appear contradictory to participants who are being emailed the survey using their name. Further reassurance may be needed. | Suggested that this was in the participant information sheet not in the invitation. HB argued that participants were saying that the it made them uncertain about whether what they were saying truly would be anonymous. | Remove | Voted, n = 11 (Only 9% voted to include). | Out |
| Producing and user testing your invitation | 35 | Co-produce your study with patients and the public. | Change the word 'study' to 'invitation' and clarify that PPI is a first step, consider having it as the first recommendation. | Adjust wording and move to the beginning of the recommendations | All agreed to action, no voting | In |
|  | 36 | User-test or pilot your study invitation with your target audience (ensuring they are a diverse group and not research-aware patient research partners). Do this even if patient research partners have been involved in the design of your study, as patient research partners are often familiar with medical or research terms. | Change the wording of this: Do this even if patient research partners have been involved in the design of your 'invitation' not 'study'. | Adjust wording | All agreed to action, no voting | In |
